# Variability of Phenological Behaviours of Wild Fruit Tree Species Based on Discriminant Analysis

**DOI:** 10.3390/plants11010045

**Published:** 2021-12-24

**Authors:** Sina Cosmulescu, Dragoș Ștefănescu, Ana-Maria Stoenescu

**Affiliations:** 1 Department of Horticulture & Food Science, Horticulture Faculty, University of Craiova, A.I. Cuza Street 13, 200585 Craiova, Romania; 2 Department of Biology & Environmental Engineering, Horticulture Faculty, University of Craiova, A.I. Cuza Street 13, 200585 Craiova, Romania; anamaria.stoenescu@edu.ucv.ro

**Keywords:** discriminant analysis, phenology, variability, wild species

## Abstract

Vegetation phenology is considered an important biological indicator in understanding the behaviour of ecosystems and how it responds to environmental cues. The aim of this paper is to provide information on the variability of phenological behaviours based on discriminant analysis using the R software package with the following libraries: ggplot2, heplots, candisc, MASS, car, and klaR. Three phenological phases were analysed with eight wild fruit tree species from a forest ecosystem in the southwestern part of Romania (44°05′19.5” N 23°54′03.5” E). It was found that there is a large and very large variability for the “bud burst” phenophase, medium and low for “full flowering”, and reduced for the “all petals fallen” phenophase. For the analyzed data, the discriminant analysis model has high accuracy (accuracy: 0.9583; 95% CI: (0.7888, 0.9989). Partition plots show the results of “full flowering” and “all petals fallen” as a function of the “bud burst” of pockmarks when separated into eight clusters and eight clusters of “full flowering” as a function of “all petals fallen”. The differences observed, from a phenological point of view, are not only due to the different cold requirements of these species but also to the temperatures during the spring.

## 1. Introduction

Phenology is the science of recurring events in nature represented by the stages of development of the species each year. Plant phenology is strongly controlled by climate and has, therefore, become one of the most widely used bioindicators of climate change, an indicator preferred by scientists because recorded data provide a high temporal resolution of changes occurred, meaning that it can provide useful data regarding the onset and length of each growth stage. Climate influences plant phenological traits, thus playing a key role in defining the geographical range of crops [1]. Uncovering the impacts of climate change has been the major focus of biodiversity research [2]. Thus, numerous analyses have demonstrated the onset of spring-specific events much earlier, respectively, which is an extension of the growing season [3,4,5]. Many phenological events, such as budding, flowering, and leaf occurrence; the timing of insect occurrence; or the arrival of migratory birds have advanced in response to recent climate change [6]. Numerous studies have documented the effects of recent climate change on plant phenology in a wide range of taxa [7,8,9,10,11,12]. Plant phenology affects the structure and function of terrestrial ecosystems and determines vegetation feedback to the climate system by altering carbon, water, and energy fluxes between the vegetation and near-surface atmosphere [13]. There is also great interest in how these phenological changes can affect ecosystem processes and services [14]. Integrating climate change into ecosystem service assessments and natural resource management is still a major challenge [15]. Many phenological observations, including long time series which are a valuable source of information for climate change-impact studies, were made in the context of diverse applications of phenology (e.g., agrometeorology and fundamental ecological research) [16]. Due to the fact that vegetation phenology is considered an important biological indicator in understanding the behaviour of ecosystems and how it responds to environmental cues [17], the aim of this paper is to provide information on the variability of phenological behaviours of wild species based on discriminant analysis.

## 2. Results and Discussions

Spring phenology is considered by researchers to be the most sensitive to climate change due to high temperatures and warm winters during the resting period that causes a much earlier start in vegetation to which photoperiod (time of species’ exposure to light), existing water resources and rainfall are added [16]. Based on the observations made, the variability of phenological characteristics was analyzed in eight fruit species identified in spontaneous flora. In order to determine the changes occurring during some phenophases, the number of days from January 1 to the landmark stage was used. Table 1 presents the statistical processing of data obtained, over 3 years of study, regarding the phenological stages: “bud burst”, “full flowering”, and “all petals fallen”. For the “bud burst” phenophase, based on Julian dates, the order in which this phenophase occurs in the identified species was as follows: *Sambucus nigra* (40 days); *Rubus caesius* (42.67 days); *Rosa canina* (53.33 days); *Prunus spinosa* (56 days); *Malus sylvestris* (59.33 days); *Pyrus pyraster* (65 days); *Crataegus monogyna* (72.67 days); and *Crataegus pentagyna* (91.33 days). From one year to another, the order in which budding occurred is maintained, but the period of development changes in correlation with environmental factors, location, habitat, etc. According to Grime et al. [18], budding of *S. nigra* may occur earlier due to high temperatures in winter, while Atkinson and Atkinson [19] mention the reference period for the appearance of leaves in February–March and flowers in May–June, which is consistent with the data obtained in this study. Regarding the budding of *C. monogyna*, Fichtner and Wissemann [20] report the period from mid-March to April, depending on altitude and location, and the April to June period was reported for the “end of flowering period”. The results obtained within the *P. spinosa* species are in accordance with those obtained by Cosmulescu and Calusaru Gavrila [21] in Romania. Regarding the “full flowering” phenophase, taking into account the Julian date, the order in which this phenophase is triggered is as follows: *Prunus spinosa* (94.67 days); *Pyrus pyraster* (97.33 days); *Malus sylvestris* (102.67 days); *Crataegus monogyna* (123 days); *Crataegus pentagyna* (138 days); *Rosa canina* (143 days); *Sambucus nigra* (146 days); and *Rubus caesius* (179.67 days). Dönmez [22] states that hawthorn has a period of 1–2 weeks with flowers present, and *C. pentagyna* blooms later than *C. monogyna*, which confirms the results obtained in this study. Worrell et al. [23] mention the crab apple blooming in the second half of May in Scotland, much later than the data obtained in this paper (April), which supports the influence of environmental and habitat conditions on phenology. According to the study by Tooke and Battey [24] the red hawthorn blooms around 13 May (±8.4 days), the rosehip blooms around 8 June (±6.2 days), and the elderberry blooms on 4 June (±6 days, 7 days) in temperate areas.

Taking into account the same Julian date up to “all petals fallen”, the order in which this phenophase takes place in the studied species is as follows: *Prunus spinosa* (106.67 days); *Pyrus pyraster* (114 days); *Malus sylvestris* (117 days); *Crataegus monogyna* (132 days); *Crataegus pentagyna* (146.67 days); *Sambucus nigra* (158 days); *Rosa canina* (159.33 days); and *Rubus caesius* (213.67 days) (Table 1).

Regarding variability, the highest coefficient of variation for the “bud burst” phenophase was determined in *S. nigra* (30%), followed by *P. spinosa* (17.04%), *R. canina* (15.94%), and *C. monogyna* (13%). The coefficient of variation of the “full flowering” phenophase was 11.39% in *P. spinosa*, followed by *P. pyraster* (9.32%), while for “all petals fallen”, the coefficient of variation was 6.78% in *P. spinosa*, followed by *P. pyraster* (4.64%). It was found that there is a large and very large variability for the “bud burst” phenophase, medium and low for “full flowering”, and reduced variability for the “all petals fallen” phenophase (Table 1). These results offer the possibility to know the average time necessary to trigger some phenological landmarks in the studied fruit species. Šebek [25] analyzed the species *P. pyraster* regarding the moments of onset of the main phenophases (flowering, fruit ripening) in Montenegro, and the results obtained are consistent with those of this paper (the beginning of the flowering period took place between 7 April and 9 May).

**Figure 1:**
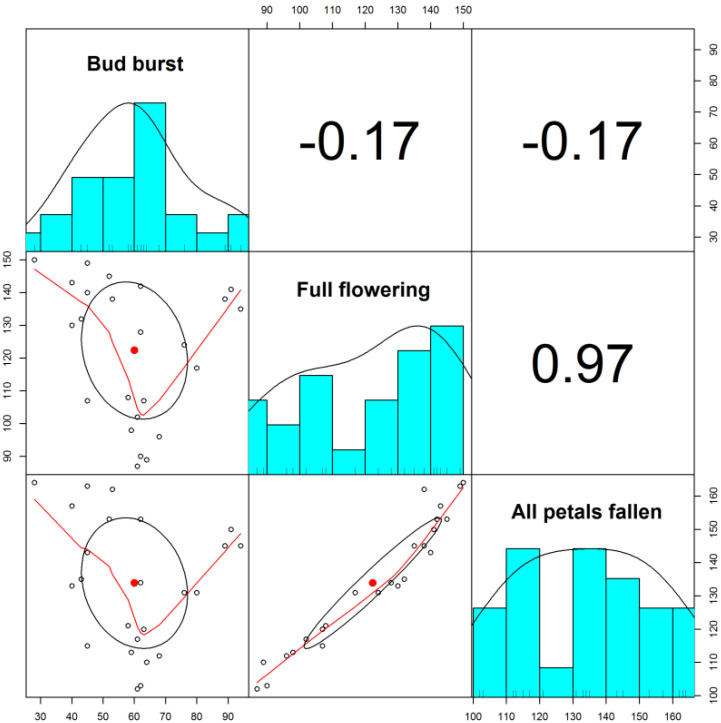
Correlation matrix for the 3 phenophases in the 8 species analysed.

The analysis of the variation of three phenological parameters by species was performed using the statistical parameter MANOVA and the discriminant analysis to highlight differences between species in terms of their response to variations in climatic parameters and to group species by phenological categories to identify groups of indicator species. Discriminant analysis seeks to find gradients of variation between groups of samples so that the variance between groups is maximized and the variance within groups is minimized along these gradients. As observed in Figure 1, there is a strong, positive correlation between “full flowering” and “all petals fallen” (correlation matrix). The correlation matrix (Figure 1) presents, in addition to the correlation values between the three variables, also the histograms of distribution of values for each variable, as well as the relationships between each pair of variables.

The variables “bud burst” and “full flowering” contribute most to the differentiation/separation of species from a phenological point of view. Following the application of MANOVA analysis, it turned out that the multivariate environments (group centroids) of the eight species have differed significantly for all four tests: Wilks test: F_(21,40)_ = 18.971, *p* < 0.0001; Wilk’s Λ = 0.001; Hotelling–Lawley test: F_(21,38)_ = 29.751, *p* < 0.0001; Hotelling–Lawley = 49.324; Pillai test: F_(21,48)_ = 11.502, *p* < 0.0001; Pillai = 2.502; Roy test: F_(7,16)_ = 93.504, *p* < 0.0001; Roy = 40.908.

Given that MANOVA analysis does not outline which variable differs significantly between species but only outlines that multivariate averages differ significantly, the differences in the means of the three variables based on ANOVA were analysed: *“bud burst”*: F_(7,16)_ = 15.793, *p* < 0.0001; “full flowering”: F_(7,16)_ = 32.107, *p* < 0.0001; and “all petals fallen”: F_(7,16)_ = 49.203, *p* < 0.0001. According to ANOVA analysis, the averages of the three variables differ significantly between the eight species (Table 1): for the “bud burst” phenophase, it is between 40 days (*S. nigra*) and 91.33 days (*C. pentagyna*); for the “full flowering” phenophase, it is between 94.67 days (*P. spinosa*) and 146 days (*S. nigra*); and for the “all petals fallen” phenophase, it is between 106.67 days (*P. spinosa*) and 159.33 days (*R. canina*).

**Figure 2:**
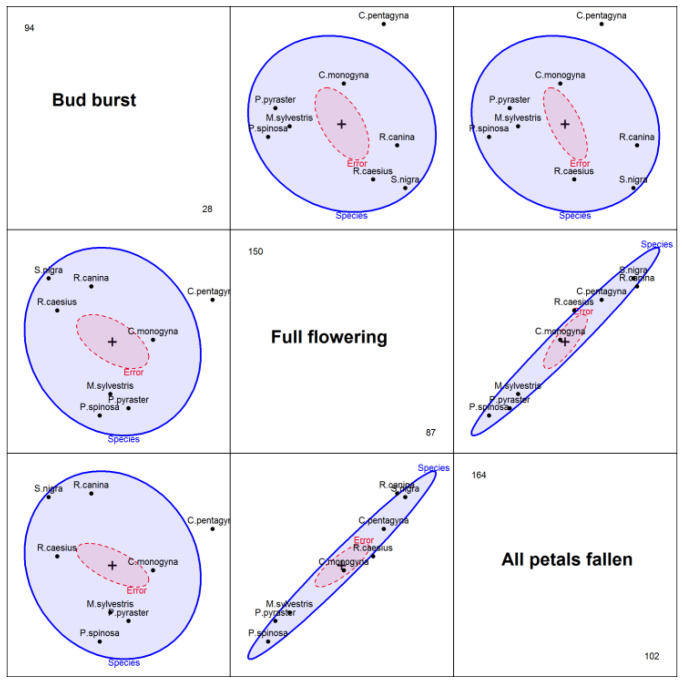
Distribution of species and averages of those distributions by pairs of variables.

Regarding the distribution of species (represented as an ellipsoid) and the averages of these distributions, on pairs of variables, this is shown in Figure 2. It is observed that the averages of these distributions, both for the phenophase “all petals fallen” and for “full flowering”, are ordered in the same manner, from *P. spinosa* to *R. canina*, which explains why the two variables are strongly correlated.

Discriminant (canonical) analysis applied to the obtained data aimed at obtaining linear combinations (canonical functions) of two or more variables that will best discriminate between a priori defined groups (Table 2).

**Figure 3:**
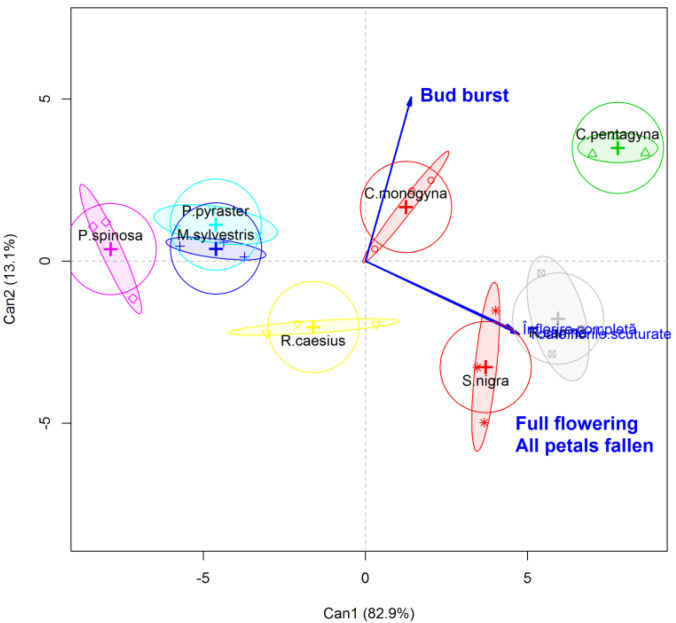
Canonical distribution of species.

As observed in Table 2, the first axis (LD1) explains most of the variation (0.829). Based on the value of correlation coefficients for the first canonical axis, “all petals fallen” (0.273) and “bud burst” (0.138) are the most important variables in discriminating/separating groups of species. “Bud burst” is the most important variable in relation to the second axis (0.122), and “full flowering” is the most important variable in relation to the third axis (0.269), and this is also visible in Figure 3.

**Figure 4:**
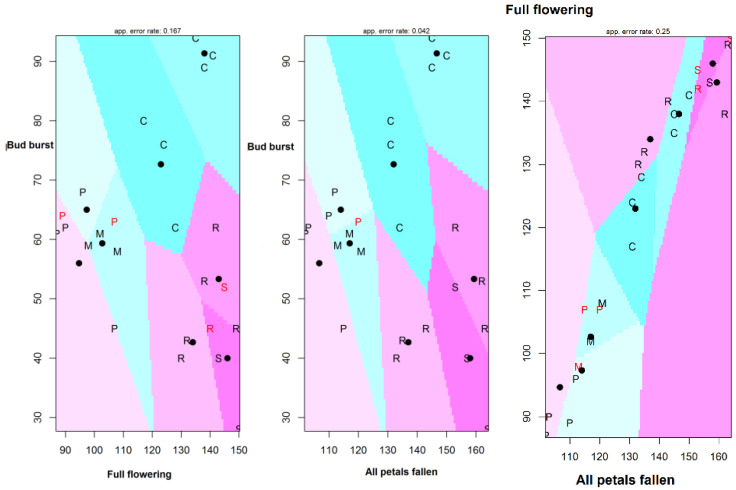
Partition plot results of “full flowering”, “all petals fallen” and “bud burst”.

*R. canina*, *S. nigra* and *C. pentagyna* are species that shake their flowers at the latest term (after several days). *C. pentagyna* also buds the latest, and *S.nigra* buds the earliest. Discriminant analysis (DF) is a predictive classification, which assigns an observation to a group starting from classification rules derived from previously classified observations. For the analyzed data, the DF model has a high accuracy (accuracy: 0.9583; 95% CI: (0.7888, 0.9989); no information rate: 0.1667; *p* < 0.0001; Kappa: 0.9524). Partition plot show the results of “full flowering” and “all petals fallen” as a function of the “bud burst” of pockmarks when separated into eight clusters and eight clusters of “full flowering” as a function of “all petals fallen” (Figure 4).

## 3. Materials and Methods

Analysis included phenological data for the period 2019–2021 from eight wild fruit tree species (*Crataegus monogyna*, *Crataegus pentagyna*, *Pyrus pyraster*, *Malus sylvestris*, *Prunus spinosa*, *Rosa canina*, *Sambucus nigra*, and *Rubus caesius*) from a forest ecosystem in the southwestern part of Romania (44°05′19.5” N 23°54′03.5” E). The Bratovoesti forest is included in the perimeter of the habitat of community interest. 91F0 mixed meadow forests of *Quercus robur, Ulmus laevis* and *Ulmus minor*, and *Fraxinus excelsior* or *Fraxinus angustifolia* along the great rivers—Ulmenion minoris. It is also part of the protected area network Natura 2000 and ROSCI0045 Jiu Corridor and has an area of 1293.6 ha. The forest ecosystem borders at the north with the Georocel stream, Bratovoesti commune, and Jiu river; at the west with Jiu river; at the east with national road DN55 Craiova-Bechet; and at the south with Jiu river and Rojişte commune. The altitude is between approximately 50 m to 80 m above sea level. The forest floor consists mostly of *Populus canadensis*, *Robinia pseudoacacia*, *Fraxinus* sp., *Quercus* sp., *Alnus glutinosa*, *Populus alba*, and *Tillia* sp. and various hard and soft essences. From the climatic point of view, the natural environment in which the research was carried out falls within the type of continental temperate climate, with rainfall of approximately 500 mm/year (a maximum of rainfall in May and June, and the driest months August and September) and with a temperature amplitude of over 25 °C [26], with very hot summers (with a maximum temperature of 38 °C) and cold winters (with an absolute minimum temperature of −27 °C). The first frost occurs after 25 October, and the last frost occurs in the first decade of April, resulting in an interval of 200 days/year without frost. The nearest weather station is located 25 km away from the site. By analysing climatic data over the 3 years of study (Table 3), the year 2021 presented much lower average monthly temperatures over the calendar period of February, March, and April and heavy rainfall in January, February, April, and May compared to 2019 and 2020. These factors contribute to the development of vegetation phenophases.

The Bratovoesti forest is located on loess, which resulted in the formation of alluvial soils and reddish brown luvic soils. At the same time, on the banks of the Jiu River on the sandy rocks, superficial soils were poor in mineral substances, respectively, and protosols were formed. The Jiu river crosses the Bratovoeşti forest on the western side from north to south, and the Georocel stream enters a small area through the northern part of the forest. Moreover, the presence of groundwater influenced the spread of soils and the formation of vegetation. Due to the shallow groundwater, many wetlands have formed with an important role for the biodiversity of the ecosystem.

Phenological observations and recordings consisted in field trips at intervals of 2–3 days and were performed according to BBCH stages. Three phenological phases were analyzed: “bud burst” (53 BBCH) with visible green leaf tips enclosing flowers, “full flowering” (65 BBCH) when at least 50% of flowers open, and “all petals fallen” (69 BBCH) which marks the end of flowering period. Ten mature genotypes from each species, located in full sun exposure were monitored and observed. Four shoots following the four compass directions were selected and marked at the base and at the same height (for all ten genotypes) depending on species [27]. The observations of the marked shoots were recorded and converted to Julian dates for the purpose of quantitative analysis. The data obtained from observations were statistically processed using descriptive statistics. For the analysis of the variability of phenological characteristics, the MANOVA parameter was used and discriminant analysis was performed using the R software package with the following libraries: ggplot2, heplots, candisc, MASS, car, and klaR.

## 4. Conclusions

In conclusion, spontaneous fruiting species are sensitive to climatic factors and sudden temperature changes causing precocity in the onset of phenophases or their delay. Photoperiod is another important factor in the development of landmarks through the process of photosynthesis along with the amount of rainfall that sometimes prolongs a certain growing season. The BBCH scale is a simple method of observing the annual cycle of fruit species from budding to resting. The differences observed, from a phenological point of view, are not only due to the different cold requirements of these species but also due to the temperatures during the spring. The high temperature of spring causes a shorter period for flowering and the “full flowering” phenophase lasts only a few days for the entire assortment of species. The MANOVA parameter and discriminant analysis are useful tools in evaluating the variability of phenological characteristics. They can provide quality information and explanations regarding the behavior of fruit species development stages as well as correlations between species and between different phenological growth stages.

## Figures and Tables

**Table 1:** Descriptive statistics of the number of days (Julian date) necessary for the development of the main spring phenological stages.

Species	Descriptive Statistics	“Bud Burst”	“Full Flowering”	“All Petals Fallen”
*Malus sylvestris*	Mean ± SD	59.33 ± 1.53	102.67 ± 5.03	117.00 ± 4.00
	Variation range	58–61	98–108	113–121
	CV%	2.58	4.90	3.42
*Pyrus pyraster*	Mean ± SD	65.00 ± 2.65	97.33 ± 9.07	114.00 ± 5.29
	Variation range	63–68	89–107	110–120
	CV%	4.08	9.32	4.64
*Crataegus monogyna*	Mean ± SD	72.67 ± 9.45	123.00 ± 5.57	132.00 ± 1.73
	Variation range	62–80	117–128	131–134
	CV	13.00	4.53	1.31
*Crataegus pentagyna*	Mean ± SD	91.33 ± 2.52	138.00 ± 3.00	146.67 ± 2.89
	Variation range	89–94	135–141	145–150
	CV%	2.76	2.17	1.97
*Rosa canina*	Mean ± SD	53.33 ± 8.50	143.00 ± 5.57	159.33 ± 5.51
	Variation range	45–62	138–149	153–163
	CV%	15.94	3.90	3.46
*Rubus caesius*	Mean ± SD	42.67 ± 2.52	179.67 ± 3.51	213.67 ± 4.51
	Variation range	40–45	176–183	209–218
	CV%	5.91	1.95	2.11
*Sambucus nigra*	Mean ± SD	40.00 ± 12.00	146.00 ± 3.61	158.00 ± 5.57
	Variation range	28–52	143–150	153–164
	CV%	30.00	2.47	3.52
*Prunus spinosa*	Mean ± SD	56.00 ± 9.54	94.67 ± 10.79	106.67 ± 7.23
	Variation range	45–62	87–107	102–115
	CV%	17.04	11.39	6.78

SD = standard deviation; CV% = coefficient of variation.

**Table 2:** Structural coefficients (correlation) between each variable and two canonical functions.

Phenology Stage	* LD1 (0.829)	LD2 (0.131)	LD3 (0.039)
“bud burst”	0.138	0.122	−0.003
“full flowering”	−0.004	0.024	0.269
“all petals fallen”	0.273	−0.056	−0.276

* LD = linear discriminant function.

**Table 3:** Climatic data for the three years of study (meteocraiova.ro).

Climate Data	Year	January	February	March	April	May	June
Average temperature (°C)	2019	−3.39	5.36	11.72	13.67	17.05	24.76
	2020	2.96	7.5	9.61	14.25	18.58	23.57
	2021	3.77	5.2	7.33	11.41	18.61	23.07
Total rainfall (mm)	2019	64.5	26.7	2.5	23.1	22.2	85
	2020	1	8.6	124.4	5.6	69.6	33.6
	2021	67.6	35.2	56	41.2	101.8	49.4

## Data Availability

Not applicable.

## References

[1] Vanalli C., Casagrandi R., Gatto M., Bevacqua D. (2021). Shifts in the thermal niche of fruit trees under climate change: The case of peach cultivation in France. Agric. For. Meteorol..

[2] Dahal N., Lamichhaney S., Kumar S. (2021). Climate change impacts on himalayan biodiversity: Evidence-based perception and current approaches to evaluate threats under climate change. J. Indian Inst. Sci..

[3] Menzel A., Sparks T.H., Estrella N., Koch E., Aasa A., Ahas R., Alm-Kübler K., Bissolli P., Braslavská O.G., Briede A. (2006). European phenological response to climate change matches the warming pattern. Glob. Chang. Biol..

[4] Cosmulescu S., Baciu A., Cichi M., Gruia M. (2010). The effect of climate changes on phenological phases in plum tree Prunus domestica in south-western Romania. South-West J. Hortic. Biol. Environ..

[5] Cosmulescu S., Baciu A., Gruia M. (2015). Influence of climatic factors on the phenology spring in Southern Oltenia Romania. J. Hortic. Sci. Biotechnol..

[6] Gordo O., Sanz J.J. (2005). Phenology and climate change: A long-term study in a Mediterranean locality. Oecologia.

[7] Cosmulescu S., Gruia M. (2016). Climatic variability in Craiova Romania and its impacts on fruit orchards. South-West J. Hortic. Biol. Environ..

[8] Cosmulescu S., Bîrsanu Ionescu M. (2018). Phenological calendar in some walnut genotypes grown in Romania and its correlations with air temperature. Int. J. Climatol..

[9] Paltineanu C., Chitu E. (2020). Climate change impact on phenological stages of sweet and sour cherry trees in a continental climate environment. Sci. Hortic..

[10] Lorite I.J., Cabezas-Luque J.M., Arquero O., Gabaldón-Leal C., Santos C., Rodríguez A., Lovera M. (2020). The role of phenology in the climate change impacts and adaptation strategies for tree crops: A case study on almond orchards in Southern Europe. Agric. For. Meteorol..

[11] Fraga H., Santos J.A. (2021). Assessment of climate change impacts on chilling and forcing for the main fresh fruit regions in Portugal. Front. Plant Sci..

[12] Santos J.A., Fraga H., Malheiro A.C., Moutinho-Pereira J., Dinis L.T., Correia C., Schultz H.R. (2020). A review of the potential climate change impacts and adaptation options for European viticulture. Appl. Sci..

[13] Fu Y., Li X., Zhou X., Geng X., Guo Y., Zhang Y. (2020). Progress in plant phenology modeling under global climate change. Sci. China Earth Sci..

[14] Markkula I., Turunen M., Rasmus S. (2019). A review of climate change impacts on the ecosystem services in the Saami Homeland in Finland. Sci. Total Environ..

[15] Yang H., Gou X., Yin D. (2021). Response of biodiversity, ecosystems, and ecosystem services to climate change in China: A Review. Ecologies.

[16] Badeck F.W., Bondeau A., Böttcher K., Doktor D., Lucht W., Schaber J., Sitch S. (2004). Responses of spring phenology to climate change. New Phytol..

[17] Caparros-Santiago J.A., Rodriguez-Galiano V., Dash J. (2021). Land surface phenology as indicator of global terrestrial ecosystem dynamics: A systematic review. ISPRS J. Photogramm. Remote Sens..

[18] Grime J.P., Hodgson J.G., Hunt R. (1988). Comparative Plant Ecology: A Functional Approach to Common British Species. (Agrostis spp.).

[19] Atkinson M.D., Atkinson E. (2002). *Sambucus nigra* L.. J. Ecol..

[20] Fichtner A., Wissemann V. (2021). Biological flora of the British isles: Crataegus monogyna. J. Ecol..

[21] Cosmulescu S., Calusaru Gavrila F. (2020). Influence of temperature on blackthorn (*Prunus spinosa* L.) phenophases in spring season. J. Agric. Meteorol..

[22] Dönmez A.A. (2004). The genus Crataegus, L. (Rosaceae) with special reference to hybridisation and biodiversity in Turkey. Turk. J. Bot..

[23] Worrell R., Ruhsam M., Renny J., Jessop W., Findlay G. (2020). Scotland’s native wild apple–Malus sylvestris: Genetic issues and conservation. Scott. For..

[24] Tooke F., Battey N.H. (2010). Temperate flowering phenology. J. Exp. Bot..

[25] Šebek G. (2019). The phenological and pomological traits of selected genotypes of wild pear [*Pyrus pyraster* (L.) Du Roi] important for the production of generative rootstocks. Acta Sci. Pol. Hortorum Cultus.

[26] Cojoacă F.D., Niculescu M. (2018). Diversity, distribution and ecology of the forest natural habitats in the Bratovoești Forest, Dolj County. Sci. Pap. Ser. A Agron..

[27] El Yamani M., Boussakouran A., Rharrabti Y. (2019). Codification and description of almond (*Prunus dulcis*) vegetative and reproductive phenology according to the extended BBCH scale. Sci. Hortic..

